# Supplementary material for: Heritability informed power optimization (HIPO) leads to enhanced detection of genetic associations across multiple traits
Source: PLoS Genet. 2018 Oct 5;14(10):e1007549. doi: 10.1371/journal.pgen.1007549 (PMC6192650; doi:10.1371/journal.pgen.1007549)
Supplement: S1 Table — (PDF) [file pgen.1007549.s001.pdf]

S1 Table. Summary of simulation settings.

<table>
<tr><th></th><th>Genetic covariance matrix</th><th>Phenotypic covariance matrix</th><th>Causal SNPs</th><th>Population stratification</th><th>Sample overlap</th></tr>
<tr><td colspan="6">1. Blood lipids</td></tr>
<tr><td>1a</td><td rowspan="2">$\Sigma_{g1} = h^2_{max}\begin{pmatrix} 0.87 & -0.04 & 0.30 & 0.85 \\ -0.04 & 1.00 & -0.62 & 0.18 \\ 0.30 & -0.62 & 0.93 & 0.30 \\ 0.85 & 0.18 & 0.30 & 0.95 \end{pmatrix}$</td><td rowspan="4">$\begin{pmatrix} 1.00 & -0.10 & 0.21 & 0.86 \\ -0.10 & 1.00 & -0.36 & 0.12 \\ 0.21 & -0.36 & 1.00 & 0.32 \\ 0.86 & 0.12 & 0.32 & 1.00 \end{pmatrix}$</td><td rowspan="2">Same set of ~12K across all traits with effect sizes generated from distribution $N(0, \frac{\Sigma_{g1}}{12K})$.</td><td>No</td><td rowspan="3">All traits are measured on the same set of subjects.</td></tr>
<tr><td>1b</td><td>Yes</td></tr>
<tr><td>1c</td><td>$0.5\Sigma_{g1} + 0.5diag(\Sigma_{g1})$
$= h^2_{max}\begin{pmatrix} 0.87 & -0.02 & 0.15 & 0.43 \\ -0.02 & 1.00 & -0.31 & 0.09 \\ 0.15 & -0.31 & 0.93 & 0.15 \\ 0.43 & 0.09 & 0.15 & 0.95 \end{pmatrix}$
Same heritability as 1a, 1b and 1d, half of the genetic covariance.</td><td>a) ~6K causal for all traits with effect size generated from $N(0, \frac{\Sigma_{g1}}{12K})$;
b) ~6K only causal for trait 1 and 4 with effect size generated from $N(0, \frac{\Sigma_{g1}^{1,4}}{12K})$;
c) ~6K only causal for trait 2 and 3 with effect size generated from $N(0, \frac{\Sigma_{g1}^{2,3}}{12K})$.</td><td>No</td></tr>
<tr><td>1d</td><td>$\Sigma_{g1} = h^2_{max}\begin{pmatrix} 0.87 & -0.04 & 0.30 & 0.85 \\ -0.04 & 1.00 & -0.62 & 0.18 \\ 0.30 & -0.62 & 0.93 & 0.30 \\ 0.85 & 0.18 & 0.30 & 0.95 \end{pmatrix}$</td><td>Same set of ~12K across all traits with effect sizes generated from distribution $N(0, \frac{\Sigma_{g1}}{12K})$.</td><td>No</td><td>Trait 1 and 4 measured on $\frac{350}{489}N$ subjects, trait 2 and 3 measured on $\frac{339}{489}N$, with $\frac{200}{489}N$ overlap.</td></tr>
<tr><td colspan="6">2. Psychiatric diseases</td></tr>
<tr><td>2a</td><td rowspan="2">$\Sigma_{g2} = h^2_{max}\begin{pmatrix} 0.69 & 0.02 & 0.12 \\ 0.02 & 0.88 & 0.63 \\ 0.12 & 0.63 & 1.00 \end{pmatrix}$</td><td rowspan="4">$\begin{pmatrix} 1.00 & 0.01 & 0.00 \\ 0.01 & 1.00 & 0.01 \\ 0.00 & 0.01 & 1.00 \end{pmatrix}$</td><td rowspan="2">Same set of ~12K across all traits with effect sizes generated from distribution $N(0, \frac{\Sigma_{g2}}{12K})$.</td><td>No</td><td rowspan="3">All traits are measured on the same set of subjects.</td></tr>
<tr><td>2b</td><td>Yes</td></tr>
<tr><td>2c</td><td>$0.5\Sigma_{g2} + 0.5\, diag(\Sigma_{g2})$
$= h^2_{max}\begin{pmatrix} 0.69 & 0.01 & 0.06 \\ 0.01 & 0.88 & 0.32 \\ 0.06 & 0.32 & 1.00 \end{pmatrix}$
Same heritability as 2a, 2b and 2d, half of the genetic covariance.</td><td>a) ~6K shared by all traits with effect size generated from $N(0, \frac{\Sigma_{g2}}{12K})$;
b) ~6K only causal for trait 1 with effect size generated from $N(0, \frac{0.69h^2_{max}}{12K})$;
c) ~6K only causal for trait 2 with effect size generated from $N(0, \frac{0.88h^2_{max}}{12K})$;
d) ~6K only causal for trait 3 and with effect size generated from $N(0, \frac{h^2_{max}}{12K})$.</td><td>No</td></tr>
<tr><td>2d</td><td>$\Sigma_{g2} = h^2_{max}\begin{pmatrix} 0.69 & 0.02 & 0.12 \\ 0.02 & 0.88 & 0.63 \\ 0.12 & 0.63 & 1.00 \end{pmatrix}$</td><td>Same set of ~12K across all traits with effect sizes generated from distribution $N(0, \frac{\Sigma_{g2}}{12K})$.</td><td>No</td><td>Trait 1 and 2 measured on $\frac{350}{489}N$ subjects, trait 3 measured on $\frac{339}{489}N$, with $\frac{200}{489}N$ overlap.</td></tr>
<tr><td colspan="6">3. Ten correlated traits</td></tr>
<tr><td>3</td><td>$\Sigma_{g3} =$
$h^2_{max}\begin{pmatrix} 1 & 0.6 & \dots & 0.6 & 0.2 & 0.2 & \dots & 0.2 \\ 0.6 & 1 & \dots & 0.6 & 0.2 & 0.2 & \dots & 0.2 \\ \dots & \dots & \dots & \dots & \dots & \dots & \dots & \dots \\ 0.6 & 0.6 & \dots & 1 & 0.2 & 0.2 & \dots & 0.2 \\ 0.2 & 0.2 & \dots & 0.2 & 1 & 0.6 & \dots & 0.6 \\ 0.2 & 0.2 & \dots & 0.2 & 0.6 & 1 & \dots & 0.6 \\ \dots & \dots & \dots & \dots & \dots & \dots & \dots & \dots \\ 0.2 & 0.2 & \dots & 0.2 & 0.6 & 0.6 & \dots & 1 \end{pmatrix}$</td><td>$\begin{pmatrix} 1 & 0.4 & \dots & 0.4 & 0.3 & 0.3 & \dots & 0.3 \\ 0.4 & 1 & \dots & 0.4 & 0.3 & 0.3 & \dots & 0.3 \\ \dots & \dots & \dots & \dots & \dots & \dots & \dots & \dots \\ 0.4 & 0.4 & \dots & 1 & 0.3 & 0.3 & \dots & 0.3 \\ 0.3 & 0.3 & \dots & 0.3 & 1 & 0.4 & \dots & 0.4 \\ 0.3 & 0.3 & \dots & 0.3 & 0.4 & 1 & \dots & 0.4 \\ \dots & \dots & \dots & \dots & \dots & \dots & \dots & \dots \\ 0.3 & 0.3 & \dots & 0.3 & 0.4 & 0.4 & \dots & 1 \end{pmatrix}$</td><td>Same set of ~12K across all traits with effect sizes generated from distribution $N(0, \frac{\Sigma_{g3}}{12K})$.</td><td>No</td><td>All traits are measured on the same set of subjects.</td></tr>
</table>

For a matrix $A = \{a_{kl}\}$, we denote by $A^{i,j}$ the 2×2 submatrix $\begin{pmatrix} a_{ii} & a_{ij} \\ a_{ji} & a_{jj} \end{pmatrix}$.
