# Supplementary material for: Heritability informed power optimization (HIPO) leads to enhanced detection of genetic associations across multiple traits
Source: PLoS Genet. 2018 Oct 5;14(10):e1007549. doi: 10.1371/journal.pgen.1007549 (PMC6192650; doi:10.1371/journal.pgen.1007549)
Supplement: S2 Table — See S1 Table 1a and 1b for detailed settings and see Table 1 for type I errors of HIPO-D1. (PDF) [file pgen.1007549.s002.pdf]

S2 Table. Type I error rates for HIPO-D2 to HIPO-D4 observed in datasets simulated under covariance structure estimated from blood lipids traits. See S1 Table 1a and 1b for detailed settings and see Table 1 for type I errors of HIPO-D1. Summary-level association statistics are simulated for 4 traits using genetic and phenotypic covariance matrices estimated from Global Lipids Genetics Consortium (GLGC) data, with the same set of causal SNPs and complete sample overlap across traits. Reported are the average of genome-wide type I error rates across 100 simulations, under significance thresholds p<0.05, p<0.01 and p<0.001.

N \ h^2_{max}	p-value threshold	0.1	0.2	0.35	0.5	0.1	0.2	0.35	0.5
**HIPO-D2**		**Without population stratification**				**With population stratification**			
**10K**	p<0.05	0.05	0.05	0.05	0.05	0.05	0.05	0.05	0.05
	p<0.01	0.01	0.01	0.01	0.01	0.01	0.01	0.01	0.01
	p<0.001	0.001	0.001	0.001	0.001	0.001	0.001	0.001	0.001
**50K**	p<0.05	0.05	0.05	0.05	0.05	0.05	0.05	0.05	0.05
	p<0.01	0.01	0.01	0.01	0.01	0.01	0.01	0.01	0.01
	p<0.001	0.001	0.001	0.001	0.001	0.001	0.001	0.001	0.001
**100K**	p<0.05	0.05	0.05	0.05	0.05	0.05	0.05	0.05	0.05
	p<0.01	0.01	0.01	0.01	0.01	0.01	0.01	0.01	0.01
	p<0.001	0.001	0.001	0.001	0.001	0.001	0.001	0.001	0.001
**500K**	p<0.05	0.05	0.05	0.049	0.049	0.05	0.05	0.049	0.049
	p<0.01	0.01	0.01	0.01	0.01	0.01	0.01	0.01	0.01
	p<0.001	0.001	0.001	0.001	0.001	0.001	0.001	0.001	0.001
**HIPO-D3**		**Without population stratification**				**With population stratification**			
**10K**	p<0.05	0.05	0.05	0.05	0.05	0.05	0.05	0.05	0.05
	p<0.01	0.01	0.01	0.01	0.01	0.01	0.01	0.01	0.01
	p<0.001	0.001	0.001	0.001	0.001	0.001	0.001	0.001	0.001
**50K**	p<0.05	0.05	0.05	0.05	0.05	0.05	0.05	0.05	0.05
	p<0.01	0.01	0.01	0.01	0.01	0.01	0.01	0.01	0.01
	p<0.001	0.001	0.001	0.001	0.001	0.001	0.001	0.001	0.001
**100K**	p<0.05	0.05	0.05	0.05	0.05	0.05	0.05	0.05	0.05
	p<0.01	0.01	0.01	0.01	0.01	0.01	0.01	0.01	0.01
	p<0.001	0.001	0.001	0.001	0.001	0.0009	0.001	0.001	0.001
**500K**	p<0.05	0.05	0.05	0.05	0.05	0.05	0.05	0.05	0.05
	p<0.01	0.01	0.01	0.01	0.01	0.01	0.01	0.01	0.01
	p<0.001	0.001	0.001	0.0009	0.001	0.001	0.001	0.0009	0.001
**HIPO-D4**		**Without population stratification**				**With population stratification**			
**10K**	p<0.05	0.049	0.049	0.05	0.05	0.049	0.049	0.05	0.05
	p<0.01	0.01	0.01	0.01	0.01	0.01	0.01	0.01	0.01
	p<0.001	0.0009	0.001	0.001	0.0009	0.0009	0.0009	0.001	0.0009
**50K**	p<0.05	0.05	0.05	0.05	0.05	0.05	0.05	0.05	0.05
	p<0.01	0.01	0.01	0.01	0.01	0.01	0.01	0.01	0.01
	p<0.001	0.0009	0.001	0.001	0.001	0.0009	0.0009	0.001	0.001
**100K**	p<0.05	0.05	0.05	0.05	0.05	0.05	0.05	0.05	0.05
	p<0.01	0.01	0.01	0.01	0.01	0.01	0.01	0.01	0.01
	p<0.001	0.001	0.0009	0.001	0.0009	0.0009	0.001	0.0009	0.0009
**500K**	p<0.05	0.05	0.05	0.05	0.05	0.05	0.05	0.05	0.05
	p<0.01	0.01	0.01	0.01	0.01	0.01	0.01	0.01	0.01
	p<0.001	0.001	0.001	0.001	0.001	0.001	0.001	0.001	0.001

h^2_{max} is the largest heritability among the individual traits.
