# Supplementary material for: Heritability informed power optimization (HIPO) leads to enhanced detection of genetic associations across multiple traits
Source: PLoS Genet. 2018 Oct 5;14(10):e1007549. doi: 10.1371/journal.pgen.1007549 (PMC6192650; doi:10.1371/journal.pgen.1007549)
Supplement: S3 Table — See S1 Table 1c and 1d for detailed settings. (PDF) [file pgen.1007549.s003.pdf]

S3 Table. Type I error rates for HIPO observed in datasets simulated under covariance structure modified from estimates of blood lipids traits allowing for partial overlap of causal SNPs and sample set across traits. See S1 Table 1c and 1d for detailed settings. Summary-level association statistics are simulated for 4 traits using genetic and phenotypic covariance matrices modified from the estimates from Global Lipids Genetics Consortium (GLGC) data, assuming partial causal SNP overlap and partial sample overlap across traits. The results for all HIPO components are listed. Reported are the average of genome-wide type I error rates across 100 simulations, under significance thresholds p<0.05, p<0.01 and p<0.001.

N \ h^2_{max}	p-value threshold	0.1	0.2	0.35	0.5	0.1	0.2	0.35	0.5
**HIPO-D1**		**Partial causal SNP overlap**				**Partial sample overlap**			
**10K**	p<0.05	0.051	0.051	0.05	0.05	0.051	0.051	0.051	0.051
	p<0.01	0.01	0.01	0.01	0.01	0.01	0.01	0.01	0.01
	p<0.001	0.001	0.001	0.001	0.001	0.0011	0.0011	0.0011	0.001
**50K**	p<0.05	0.05	0.05	0.05	0.051	0.051	0.051	0.05	0.05
	p<0.01	0.01	0.01	0.01	0.01	0.01	0.01	0.01	0.01
	p<0.001	0.001	0.001	0.001	0.001	0.001	0.001	0.001	0.0011
**100K**	p<0.05	0.05	0.05	0.05	0.05	0.05	0.05	0.05	0.051
	p<0.01	0.01	0.01	0.01	0.01	0.01	0.01	0.01	0.01
	p<0.001	0.001	0.001	0.001	0.001	0.001	0.001	0.001	0.001
**500K**	p<0.05	0.05	0.051	0.051	0.052	0.051	0.051	0.05	0.051
	p<0.01	0.01	0.01	0.01	0.011	0.01	0.01	0.01	0.011
	p<0.001	0.001	0.001	0.0011	0.0011	0.001	0.0011	0.001	0.0011
**HIPO-D2**		**Partial causal SNP overlap**				**Partial sample overlap**			
**10K**	p<0.05	0.05	0.05	0.05	0.05	0.05	0.05	0.05	0.05
	p<0.01	0.01	0.01	0.01	0.01	0.01	0.01	0.01	0.01
	p<0.001	0.001	0.001	0.001	0.001	0.001	0.001	0.001	0.001
**50K**	p<0.05	0.05	0.05	0.05	0.05	0.05	0.05	0.05	0.05
	p<0.01	0.01	0.01	0.01	0.01	0.01	0.01	0.01	0.01
	p<0.001	0.001	0.001	0.001	0.001	0.001	0.001	0.001	0.001
**100K**	p<0.05	0.05	0.05	0.05	0.05	0.05	0.05	0.05	0.05
	p<0.01	0.01	0.01	0.01	0.01	0.01	0.01	0.01	0.01
	p<0.001	0.001	0.001	0.001	0.001	0.001	0.001	0.001	0.001
**500K**	p<0.05	0.05	0.05	0.05	0.05	0.05	0.05	0.049	0.049
	p<0.01	0.01	0.01	0.01	0.01	0.01	0.01	0.01	0.01
	p<0.001	0.001	0.001	0.001	0.001	0.001	0.001	0.001	0.001
**HIPO-D3**		**Partial causal SNP overlap**				**Partial sample overlap**			
**10K**	p<0.05	0.05	0.05	0.05	0.05	0.05	0.05	0.05	0.05
	p<0.01	0.01	0.01	0.01	0.01	0.01	0.01	0.01	0.01
	p<0.001	0.001	0.001	0.001	0.001	0.001	0.001	0.001	0.001
**50K**	p<0.05	0.05	0.05	0.05	0.05	0.05	0.05	0.05	0.05
	p<0.01	0.01	0.01	0.01	0.01	0.01	0.01	0.01	0.01
	p<0.001	0.001	0.001	0.001	0.001	0.001	0.001	0.001	0.001
**100K**	p<0.05	0.05	0.05	0.049	0.05	0.05	0.05	0.05	0.05
	p<0.01	0.01	0.01	0.01	0.01	0.01	0.01	0.01	0.01
	p<0.001	0.001	0.001	0.001	0.001	0.001	0.001	0.001	0.001
**500K**	p<0.05	0.05	0.05	0.05	0.05	0.05	0.05	0.05	0.05
	p<0.01	0.01	0.01	0.01	0.01	0.01	0.01	0.01	0.01
	p<0.001	0.001	0.001	0.001	0.001	0.001	0.001	0.001	0.001
**HIPO-D4**		**Partial causal SNP overlap**				**Partial sample overlap**			
**10K**	p<0.05	0.049	0.049	0.05	0.05	0.049	0.049	0.049	0.05
	p<0.01	0.01	0.01	0.01	0.01	0.01	0.01	0.01	0.01
	p<0.001	0.001	0.001	0.001	0.0009	0.0009	0.001	0.001	0.001
**50K**	p<0.05	0.05	0.05	0.05	0.05	0.05	0.05	0.05	0.05

	p<0.01	0.01	0.01	0.01	0.01	0.01	0.01	0.01	0.01
	p<0.001	0.001	0.001	0.001	0.001	0.001	0.001	0.001	0.001
**100K**	p<0.05	0.05	0.05	0.05	0.05	0.05	0.05	0.05	0.05
	p<0.01	0.01	0.01	0.01	0.01	0.01	0.01	0.01	0.01
	p<0.001	0.0009	0.0009	0.0009	0.0009	0.001	0.001	0.001	0.001
**500K**	p<0.05	0.05	0.05	0.05	0.049	0.05	0.05	0.05	0.05
	p<0.01	0.01	0.01	0.01	0.01	0.01	0.01	0.01	0.01
	p<0.001	0.001	0.001	0.0009	0.0009	0.001	0.001	0.001	0.001

h^2_{max} is the largest heritability among the individual traits.
