# Supplementary material for: Heritability informed power optimization (HIPO) leads to enhanced detection of genetic associations across multiple traits
Source: PLoS Genet. 2018 Oct 5;14(10):e1007549. doi: 10.1371/journal.pgen.1007549 (PMC6192650; doi:10.1371/journal.pgen.1007549)
Supplement: S4 Table — See S1 Table 2a and 2b for detailed settings. (PDF) [file pgen.1007549.s004.pdf]

S4 Table. Type I error rates for HIPO observed in datasets simulated under covariance structure estimated from studies of psychiatric diseases. See S1 Table 2a and 2b for detailed settings. Summary-level association statistics are simulated for 3 traits using genetic and phenotypic covariance matrices for autism spectrum disorder, bipolar disorder and schizophrenia estimated from Psychiatric Genomics Consortium (PGC) data. The results for the most heritable trait and HIPO-D1 to HIPO-D3 are listed. Reported are the average of genome-wide type I error rates across 100 simulations, under significance thresholds p<0.05, p<0.01 and p<0.001.

h^2_{max} / N	p-value threshold	0.1	0.2	0.35	0.5	0.1	0.2	0.35	0.5
**Most heritable trait**		**Without population stratification**				**With population stratification**			
**10K**	p<0.05	0.051	0.051	0.05	0.051	0.051	0.051	0.051	0.051
	p<0.01	0.01	0.01	0.01	0.01	0.01	0.01	0.01	0.01
	p<0.001	0.0011	0.0011	0.0011	0.0011	0.0011	0.0011	0.0011	0.0011
**50K**	p<0.05	0.05	0.051	0.05	0.051	0.051	0.051	0.051	0.051
	p<0.01	0.01	0.01	0.01	0.01	0.01	0.01	0.01	0.01
	p<0.001	0.0011	0.0011	0.0011	0.0011	0.0011	0.0011	0.0011	0.0011
**100K**	p<0.05	0.051	0.05	0.051	0.05	0.051	0.051	0.051	0.051
	p<0.01	0.01	0.01	0.01	0.01	0.01	0.011	0.011	0.011
	p<0.001	0.0011	0.0011	0.0011	0.0011	0.0011	0.0011	0.0011	0.0011
**500K**	p<0.05	0.05	0.051	0.05	0.051	0.055	0.055	0.055	0.055
	p<0.01	0.01	0.01	0.01	0.01	0.012	0.012	0.012	0.012
	p<0.001	0.0011	0.0011	0.0011	0.0011	0.0013	0.0013	0.0013	0.0013
**HIPO-D1**		**Without population stratification**				**With population stratification**			
**10K**	p<0.05	0.051	0.05	0.05	0.05	0.051	0.05	0.05	0.05
	p<0.01	0.01	0.01	0.01	0.01	0.01	0.01	0.01	0.01
	p<0.001	0.001	0.001	0.001	0.001	0.001	0.001	0.001	0.001
**50K**	p<0.05	0.05	0.05	0.05	0.05	0.05	0.05	0.05	0.05
	p<0.01	0.01	0.01	0.01	0.01	0.01	0.01	0.01	0.01
	p<0.001	0.001	0.001	0.001	0.001	0.001	0.001	0.001	0.001
**100K**	p<0.05	0.05	0.05	0.05	0.05	0.05	0.05	0.05	0.05
	p<0.01	0.01	0.01	0.01	0.01	0.01	0.01	0.01	0.01
	p<0.001	0.001	0.001	0.001	0.001	0.001	0.001	0.001	0.001
**500K**	p<0.05	0.05	0.05	0.05	0.051	0.05	0.05	0.051	0.051
	p<0.01	0.01	0.01	0.01	0.01	0.01	0.01	0.01	0.01
	p<0.001	0.001	0.001	0.001	0.0011	0.001	0.001	0.001	0.0011
**HIPO-D2**		**Without population stratification**				**With population stratification**			
**10K**	p<0.05	0.05	0.05	0.05	0.05	0.05	0.05	0.05	0.05
	p<0.01	0.01	0.01	0.01	0.01	0.01	0.01	0.01	0.01
	p<0.001	0.001	0.001	0.001	0.001	0.001	0.001	0.001	0.001
**50K**	p<0.05	0.05	0.05	0.05	0.05	0.05	0.05	0.05	0.05
	p<0.01	0.01	0.01	0.01	0.01	0.01	0.01	0.01	0.01
	p<0.001	0.001	0.001	0.001	0.001	0.001	0.001	0.001	0.001
**100K**	p<0.05	0.05	0.05	0.05	0.05	0.05	0.05	0.05	0.05
	p<0.01	0.01	0.01	0.01	0.01	0.01	0.01	0.01	0.01
	p<0.001	0.001	0.0011	0.001	0.001	0.001	0.0011	0.001	0.001
**500K**	p<0.05	0.05	0.05	0.05	0.05	0.05	0.05	0.05	0.05
	p<0.01	0.01	0.01	0.01	0.01	0.01	0.01	0.01	0.01
	p<0.001	0.001	0.001	0.001	0.001	0.001	0.001	0.0011	0.001
**HIPO-D3**		**Without population stratification**				**With population stratification**			
**10K**	p<0.05	0.049	0.05	0.05	0.05	0.049	0.049	0.05	0.05
	p<0.01	0.01	0.01	0.01	0.01	0.01	0.01	0.01	0.01

	p<0.001	0.001	0.001	0.001	0.001	0.001	0.001	0.001	0.001
**50K**	p<0.05	0.05	0.05	0.05	0.05	0.05	0.05	0.05	0.05
	p<0.01	0.01	0.01	0.01	0.01	0.01	0.01	0.01	0.01
	p<0.001	0.001	0.001	0.001	0.001	0.001	0.001	0.001	0.001
**100K**	p<0.05	0.05	0.05	0.05	0.05	0.05	0.05	0.05	0.05
	p<0.01	0.01	0.01	0.01	0.01	0.01	0.01	0.01	0.01
	p<0.001	0.001	0.001	0.001	0.001	0.001	0.001	0.001	0.001
**500K**	p<0.05	0.05	0.05	0.05	0.05	0.05	0.05	0.05	0.05
	p<0.01	0.01	0.01	0.01	0.01	0.01	0.01	0.01	0.01
	p<0.001	0.001	0.001	0.001	0.001	0.001	0.001	0.001	0.001

h^2_{max} is the largest heritability among the individual traits.
