# Supplementary material for: Heritability informed power optimization (HIPO) leads to enhanced detection of genetic associations across multiple traits
Source: PLoS Genet. 2018 Oct 5;14(10):e1007549. doi: 10.1371/journal.pgen.1007549 (PMC6192650; doi:10.1371/journal.pgen.1007549)
Supplement: S6 Table — (PDF) [file pgen.1007549.s006.pdf]

**S6 Table. Percentage increase in average number of true discoveries by HIPO compared to the analysis of most heritable trait in simulation studies.** The percentage increase is calculated as 100×(average # of SNPs discovered by HIPO-D1 - average # of SNPs discovered by the most heritable trait) / (average # of SNPs discovered by the most heritable trait). The number of associated SNPs is pre-LD-clumping. The average is taken over 100 simulations.

| N \ $h^2_{max}$ | 0.1 | 0.2 | 0.35 | 0.5 | 0.1 | 0.2 | 0.35 | 0.5 |
|---|---|---|---|---|---|---|---|---|
| | **Blood lipids – without PS** | | | | **Blood lipids – with PS** | | | |
| **10K** | -75 | -9.4 | 57.1 | 69.7 | -50 | -5.6 | 69 | 55.1 |
| **50K** | 147.1 | 101.5 | 94.9 | 63.9 | 107.1 | 121.7 | 83.8 | 65.2 |
| **100K** | 114.8 | 88 | 66.5 | 57.3 | 106.1 | 82.9 | 62.2 | 52.5 |
| **500K** | 56.6 | 36.9 | 27.3 | 24.6 | 46.3 | 30.5 | 26.3 | 21.4 |
| | **Blood lipids – partial causal SNP overlap** | | | | **Blood lipids – partial sample overlap** | | | |
| **10K** | -58.3 | -21.4 | 15.6 | 92 | 125 | -69.2 | 34.8 | -2.1 |
| **50K** | 57.8 | 67.4 | 73.1 | 65.5 | 8.5 | 173.8 | 115.6 | 86.3 |
| **100K** | 75.8 | 63.4 | 50.2 | 43.7 | 127.8 | 127.8 | 85.6 | 74.2 |
| **500K** | 45.5 | 33.8 | 28.9 | 27.6 | 74 | 52.3 | 36.5 | 32.4 |
| | **Psychiatric diseases – without PS** | | | | **Psychiatric diseases – with PS** | | | |
| **10K** | 14.3 | 78.3 | 500 | 357.7 | -57.1 | 87 | 166.2 | 322.7 |
| **50K** | 487.1 | 418.4 | 292.4 | 242.8 | 294.7 | 405.4 | 290.9 | 232.7 |
| **100K** | 381.6 | 274.4 | 205.9 | 168.5 | 279.4 | 274 | 191.3 | 157.4 |
| **500K** | 170.5 | 104.1 | 75.4 | 60.5 | 131 | 86.2 | 63.3 | 51.3 |
| | **Psychiatric diseases – partial causal SNP overlap** | | | | **Psychiatric diseases – partial sample overlap** | | | |
| **10K** | -40 | -17.9 | 88.2 | -0.6 | -33.3 | -9.1 | 262.5 | 372.7 |
| **50K** | 100 | 144.8 | 105.7 | 85.7 | 437.1 | 528.4 | 310.4 | 312.8 |
| **100K** | 122.1 | 100.1 | 69.8 | 54.1 | 523.7 | 326.4 | 265.2 | 215.5 |
| **500K** | 61.3 | 34.6 | 25.7 | 24.6 | 223.2 | 135.3 | 95.9 | 78.2 |

PS: population stratification.
