# Supplementary material for: Heritability informed power optimization (HIPO) leads to enhanced detection of genetic associations across multiple traits
Source: PLoS Genet. 2018 Oct 5;14(10):e1007549. doi: 10.1371/journal.pgen.1007549 (PMC6192650; doi:10.1371/journal.pgen.1007549)
Supplement: S7 Table — (PDF) [file pgen.1007549.s007.pdf]

S7 Table. Average χ^2 for HIPO-D1 compared to those for individual traits and MTAG observed in simulation studies based on covariance structure of blood lipids. Mean (standard deviation) of the average χ^2 statistics are reported based on 100 simulations. We only list scenarios without population stratification (S1 Table 1a, 1c and 1d).

N \ h^2_{max}		0.1	0.2	0.35	0.5
	**Same causal SNPs and complete sample overlap**				
**10K**	Individual traits max	1.03 (0.01)	1.05 (0.01)	1.07 (0.01)	1.09 (0.01)
	HIPO-D1	1.03 (0.01)	1.05 (0.01)	1.07 (0.01)	1.1 (0.01)
	MTAG max	1.03 (0.01)	1.04 (0.01)	1.07 (0.01)	1.1 (0.01)
**50K**	Individual traits max	1.09 (0.01)	1.18 (0.01)	1.3 (0.02)	1.43 (0.03)
	HIPO-D1	1.1 (0.01)	1.21 (0.02)	1.36 (0.03)	1.5 (0.04)
	MTAG max	1.1 (0.01)	1.2 (0.02)	1.34 (0.03)	1.48 (0.04)
**100K**	Individual traits max	1.18 (0.01)	1.34 (0.02)	1.6 (0.04)	1.84 (0.05)
	HIPO-D1	1.2 (0.02)	1.41 (0.03)	1.71 (0.06)	2.01 (0.08)
	MTAG max	1.2 (0.02)	1.39 (0.03)	1.68 (0.06)	1.95 (0.07)
**500K**	Individual traits max	1.84 (0.05)	2.71 (0.12)	3.91 (0.16)	5.22 (0.25)
	HIPO-D1	2.01 (0.08)	3.03 (0.18)	4.48 (0.32)	6.08 (0.52)
	MTAG max	1.96 (0.07)	2.89 (0.17)	4.17 (0.28)	5.59 (0.4)
	**Partial causal SNP overlap**				
**10K**	Individual traits max	1.03 (0.01)	1.05 (0.01)	1.07 (0.01)	1.09 (0.01)
	HIPO-D1	1.03 (0.01)	1.05 (0.01)	1.08 (0.01)	1.11 (0.01)
	MTAG max	1.03 (0.01)	1.05 (0.01)	1.07 (0.01)	1.1 (0.01)
**50K**	Individual traits max	1.09 (0.01)	1.17 (0.01)	1.3 (0.02)	1.43 (0.02)
	HIPO-D1	1.11 (0.01)	1.21 (0.02)	1.37 (0.03)	1.53 (0.04)
	MTAG max	1.1 (0.01)	1.19 (0.02)	1.34 (0.03)	1.49 (0.04)
**100K**	Individual traits max	1.18 (0.01)	1.34 (0.02)	1.59 (0.04)	1.84 (0.05)
	HIPO-D1	1.21 (0.02)	1.42 (0.04)	1.72 (0.06)	2.03 (0.09)
	MTAG max	1.2 (0.02)	1.39 (0.03)	1.66 (0.05)	1.93 (0.08)
**500K**	Individual traits max	1.84 (0.05)	2.71 (0.09)	3.97 (0.17)	5.19 (0.19)
	HIPO-D1	2.04 (0.09)	3.09 (0.17)	4.69 (0.4)	6.2 (0.4)
	MTAG max	1.94 (0.08)	2.87 (0.14)	4.21 (0.28)	5.47 (0.29)
	**Partial sample overlap**				
**10K**	Individual traits max	1.03 (0.01)	1.04 (0.01)	1.05 (0.01)	1.07 (0.01)
	HIPO-D1	1.02 (0.01)	1.04 (0.01)	1.05 (0.01)	1.07 (0.01)
	MTAG max	1.02 (0.01)	1.03 (0.01)	1.05 (0.01)	1.07 (0.01)
**50K**	Individual traits max	1.07 (0.01)	1.13 (0.01)	1.22 (0.01)	1.3 (0.02)
	HIPO-D1	1.07 (0.01)	1.15 (0.01)	1.26 (0.02)	1.35 (0.03)
	MTAG max	1.07 (0.01)	1.14 (0.01)	1.25 (0.02)	1.34 (0.03)
**100K**	Individual traits max	1.13 (0.01)	1.24 (0.02)	1.42 (0.03)	1.59 (0.03)
	HIPO-D1	1.14 (0.01)	1.29 (0.02)	1.5 (0.04)	1.71 (0.05)
	MTAG max	1.14 (0.01)	1.28 (0.02)	1.48 (0.04)	1.69 (0.05)
**500K**	Individual traits max	1.59 (0.03)	2.2 (0.08)	3.04 (0.11)	3.95 (0.17)
	HIPO-D1	1.72 (0.06)	2.45 (0.13)	3.46 (0.21)	4.59 (0.35)
	MTAG max	1.69 (0.05)	2.38 (0.13)	3.32 (0.2)	4.38 (0.31)

h^2_{max} is the largest heritability among the individual traits; individual traits max: the maximum mean χ^2 among individual traits; MTAG max: the maximum mean χ^2 among all MTAG estimates.
