# Supplementary material for: Heritability informed power optimization (HIPO) leads to enhanced detection of genetic associations across multiple traits
Source: PLoS Genet. 2018 Oct 5;14(10):e1007549. doi: 10.1371/journal.pgen.1007549 (PMC6192650; doi:10.1371/journal.pgen.1007549)
Supplement: S8 Table — (PDF) [file pgen.1007549.s008.pdf]

S8 Table. Average χ^2 for HIPO-D1 compared to those for individual traits and MTAG observed in simulation studies based on covariance structure of psychiatric diseases. Mean (standard deviation) of the average χ^2 statistics are reported based on 100 simulations. We only list scenarios without population stratification (S1 Table 2a, 2c and 2d).

N \ h^2_{max}		0.1	0.2	0.35	0.5
	**Same causal SNPs and complete sample overlap**				
**10K**	Individual traits max	1.03 (0.01)	1.04 (0.01)	1.07 (0.01)	1.09 (0.01)
	HIPO-D1	1.03 (0.01)	1.05 (0.01)	1.09 (0.01)	1.13 (0.01)
	MTAG max	1.03 (0.01)	1.05 (0.01)	1.09 (0.01)	1.13 (0.01)
**50K**	Individual traits max	1.09 (0.01)	1.17 (0.02)	1.29 (0.02)	1.42 (0.03)
	HIPO-D1	1.13 (0.01)	1.27 (0.03)	1.46 (0.04)	1.65 (0.06)
	MTAG max	1.13 (0.01)	1.26 (0.03)	1.44 (0.04)	1.61 (0.06)
**100K**	Individual traits max	1.17 (0.01)	1.33 (0.02)	1.59 (0.04)	1.84 (0.05)
	HIPO-D1	1.26 (0.02)	1.52 (0.05)	1.92 (0.1)	2.32 (0.12)
	MTAG max	1.25 (0.02)	1.5 (0.05)	1.86 (0.09)	2.22 (0.11)
**500K**	Individual traits max	1.83 (0.05)	2.66 (0.11)	3.89 (0.18)	5.16 (0.24)
	HIPO-D1	2.31 (0.13)	3.61 (0.26)	5.56 (0.42)	7.51 (0.79)
	MTAG max	2.21 (0.12)	3.29 (0.22)	4.77 (0.33)	6.2 (0.53)
	**Partial causal SNP overlap**				
**10K**	Individual traits max	1.03 (0.01)	1.04 (0.01)	1.07 (0.01)	1.09 (0.01)
	HIPO-D1	1.03 (0.01)	1.04 (0.01)	1.07 (0.01)	1.1 (0.01)
	MTAG max	1.03 (0.01)	1.04 (0.01)	1.07 (0.01)	1.1 (0.01)
**50K**	Individual traits max	1.09 (0.01)	1.17 (0.01)	1.3 (0.02)	1.42 (0.03)
	HIPO-D1	1.11 (0.01)	1.21 (0.02)	1.37 (0.03)	1.53 (0.04)
	MTAG max	1.1 (0.01)	1.19 (0.02)	1.33 (0.03)	1.48 (0.04)
**100K**	Individual traits max	1.17 (0.02)	1.34 (0.02)	1.58 (0.04)	1.83 (0.06)
	HIPO-D1	1.21 (0.02)	1.42 (0.04)	1.73 (0.06)	2.04 (0.1)
	MTAG max	1.19 (0.02)	1.38 (0.03)	1.66 (0.06)	1.92 (0.08)
**500K**	Individual traits max	1.84 (0.06)	2.66 (0.1)	3.91 (0.18)	5.16 (0.25)
	HIPO-D1	2.07 (0.1)	3.08 (0.2)	4.65 (0.35)	6.25 (0.57)
	MTAG max	1.94 (0.09)	2.79 (0.15)	4.06 (0.26)	5.39 (0.38)
	**Partial sample overlap**				
**10K**	Individual traits max	1.02 (0.01)	1.03 (0.01)	1.05 (0.01)	1.06 (0.01)
	HIPO-D1	1.02 (0.01)	1.04 (0.01)	1.06 (0.01)	1.09 (0.01)
	MTAG max	1.02 (0.01)	1.04 (0.01)	1.06 (0.01)	1.09 (0.01)
**50K**	Individual traits max	1.07 (0.01)	1.12 (0.01)	1.21 (0.02)	1.29 (0.02)
	HIPO-D1	1.09 (0.01)	1.19 (0.02)	1.32 (0.03)	1.46 (0.04)
	MTAG max	1.09 (0.01)	1.18 (0.02)	1.31 (0.03)	1.43 (0.04)
**100K**	Individual traits max	1.12 (0.01)	1.23 (0.02)	1.41 (0.03)	1.58 (0.04)
	HIPO-D1	1.18 (0.02)	1.36 (0.03)	1.64 (0.06)	1.92 (0.09)
	MTAG max	1.18 (0.02)	1.35 (0.03)	1.61 (0.06)	1.86 (0.08)
**500K**	Individual traits max	1.58 (0.03)	2.15 (0.07)	3.01 (0.12)	3.89 (0.17)
	HIPO-D1	1.91 (0.08)	2.82 (0.18)	4.19 (0.27)	5.61 (0.47)
	MTAG max	1.85 (0.08)	2.65 (0.16)	3.73 (0.23)	4.8 (0.33)

h^2_{max} is the largest heritability among the individual traits; individual traits max: the maximum mean χ^2 among individual traits; MTAG max: the maximum mean χ^2 among all MTAG estimates.
