# Supplementary material for: Heritability informed power optimization (HIPO) leads to enhanced detection of genetic associations across multiple traits
Source: PLoS Genet. 2018 Oct 5;14(10):e1007549. doi: 10.1371/journal.pgen.1007549 (PMC6192650; doi:10.1371/journal.pgen.1007549)
Supplement: S9 Table — See S1 Table 2a-2d for detailed settings. (PDF) [file pgen.1007549.s009.pdf]

S9 Table. Number of truly associated independent loci discovered by HIPO, MTAG and individual trait analysis observed in datasets simulated under the covariance structure estimated from psychiatric diseases. See S1 Table 2a-2d for detailed settings. We report the average number of truly associated independent loci identified by all the individual traits/HIPO components/MTAG estimates across 100 simulations, under significance threshold $p < 5\times10^{-8}$ and LD pruning threshold $r^2 < 0.1$ and different loci required to be >0.5Mb apart.

N \ h^2_{max}		0.1	0.2	0.35	0.5	0.1	0.2	0.35	0.5
**Same causal SNPs**		**Without population stratification**				**With population stratification**			
**10K**	Individual traits	0	0	1	2	0	0	1	2
	HIPO	0	0	1	5	0	0	1	4
	MTAG	0	0	2	6	0	0	2	6
	HIPO new	0	0	1	4	0	0	1	3
	MTAG new	0	0	1	5	0	0	1	4
**50K**	Individual traits	2	17	92	248	2	17	94	251
	HIPO	5	39	181	418	4	38	183	418
	MTAG	6	47	210	473	5	46	212	472
	HIPO new	4	28	115	224	3	28	115	222
	MTAG new	5	34	131	249	4	34	130	246
**100K**	Individual traits	17	134	550	1069	18	136	552	1069
	HIPO	37	252	807	1363	36	250	788	1337
	MTAG	46	290	887	1456	45	287	865	1428
	HIPO new	27	152	347	418	26	149	329	400
	MTAG new	33	172	372	433	31	168	351	409
**500K**	Individual traits	1059	2305	3047	3389	1097	2331	3060	3394
	HIPO	1354	2459	3098	3412	1285	2404	3072	3392
	MTAG	1445	2518	3124	3421	1376	2466	3097	3404
	HIPO new	420	280	137	80	350	231	114	67
	MTAG new	433	255	103	49	350	199	77	36
		**Partial causal SNP overlap**				**Partial sample overlap**			
**10K**	Individual traits	0	0	1	2	0	0	0	1
	HIPO	0	0	1	2	0	0	0	1
	MTAG	0	0	1	3	0	0	1	2
	HIPO new	0	0	1	1	0	0	0	1
	MTAG new	0	0	1	1	0	0	1	1
**50K**	Individual traits	2	17	95	257	1	6	33	96
	HIPO	2	21	109	267	1	12	68	184
	MTAG	3	25	128	319	2	15	82	215
	HIPO new	2	14	61	123	1	9	47	116
	MTAG new	2	11	46	90	1	12	55	132
**100K**	Individual traits	18	142	568	1114	5	49	240	560
	HIPO	21	156	547	1019	12	99	410	816
	MTAG	26	186	653	1204	15	118	463	895
	HIPO new	14	84	194	238	9	66	223	347
	MTAG new	11	63	131	150	12	78	247	371
**500K**	Individual traits	1113	2411	3164	3493	551	1685	2622	3056
	HIPO	1035	2205	3007	3390	804	1924	2725	3105
	MTAG	1216	2449	3171	3497	884	2002	2770	3128
	HIPO new	246	156	72	42	344	377	217	133
	MTAG new	161	78	27	14	368	365	184	98

h^2_{max} is the largest heritability among the individual traits.
