# Supplementary material for: Heritability informed power optimization (HIPO) leads to enhanced detection of genetic associations across multiple traits
Source: PLoS Genet. 2018 Oct 5;14(10):e1007549. doi: 10.1371/journal.pgen.1007549 (PMC6192650; doi:10.1371/journal.pgen.1007549)
Supplement: S10 Table — See S1 Table part 3 for detailed settings. (PDF) [file pgen.1007549.s010.pdf]

S10 Table. Number of truly associated independent loci discovered by HIPO, MTAG and individual trait analysis from simulation studies of 10 correlated traits. See S1 Table part 3 for detailed settings. Summary level data are simulated under the same set of causal SNPs and complete sample overlap across traits. We report the average number of independent loci identified by all the individual traits/HIPO components/MTAG estimates across 100 simulations, under significance threshold $p < 5\times10^{-8}$and LD pruning threshold $r^2 < 0.1$ and different loci required to be >0.5Mb apart.

N \ h^2_{max}		**0.1**	**0.2**	**0.35**	**0.5**
**10K**	Individual traits	1	1	3	9
	HIPO	0	1	5	15
	MTAG	1	2	7	21
	HIPO new	0	1	4	14
	MTAG new	1	1	6	16
**50K**	Individual traits	9	74	358	818
	HIPO	15	125	516	1043
	MTAG	21	153	582	1122
	HIPO new	14	101	332	512
	MTAG new	16	95	274	380
**100K**	Individual traits	75	498	1479	2254
	HIPO	128	675	1693	2405
	MTAG	155	748	1745	2417
	HIPO new	105	398	558	446
	MTAG new	97	310	355	242
**500K**	Individual traits	2248	3279	3810	4110
	HIPO	2403	3341	3837	4123
	MTAG	2408	3300	3810	4109
	HIPO new	448	177	61	28
	MTAG new	238	55	12	4

PS: population stratification.
