# Supplementary material for: Heritability informed power optimization (HIPO) leads to enhanced detection of genetic associations across multiple traits
Source: PLoS Genet. 2018 Oct 5;14(10):e1007549. doi: 10.1371/journal.pgen.1007549 (PMC6192650; doi:10.1371/journal.pgen.1007549)
Supplement: S11 Table — See 1a-1d in S1 Table for detailed settings. (PDF) [file pgen.1007549.s011.pdf]

S11 Table. Number of truly associated independent loci discovered by HIPO, MTAG and individual trait analysis observed in datasets simulated under the covariance structure estimated from studies of blood lipids under alternative LD-clumping threshold. Se 1a-1d in S1 Table for detailed settings. We report the average number of truly associated loci identified by all the individual traits/HIPO components/MTAG estimates across 100 simulations, under significance threshold $p < 5\times10^{-8}$ and LD pruning threshold $r^2 < 0.1$ and different loci required to be >1Mb apart.

N \ h^2_{max}		0.1	0.2	0.35	0.5	0.1	0.2	0.35	0.5
**Same causal SNPs**		**Without population stratification**				**With population stratification**			
**10K**	Individual traits	0	1	1	3	0	0	1	3
	HIPO	0	0	1	2	0	0	1	2
	MTAG	0	1	1	4	0	0	1	4
	HIPO new	0	0	1	2	0	0	1	1
	MTAG new	0	0	1	2	0	0	1	2
**50K**	Individual traits	3	25	130	317	3	25	131	319
	HIPO	2	24	125	296	2	23	124	297
	MTAG	4	32	159	361	3	32	158	361
	HIPO new	2	12	48	83	1	12	48	86
	MTAG new	2	13	47	77	1	12	46	76
**100K**	Individual traits	25	183	622	1009	26	192	628	1018
	HIPO	23	176	590	967	24	178	584	965
	MTAG	32	220	678	1058	32	222	675	1056
	HIPO new	12	62	120	116	12	59	112	109
	MTAG new	12	59	101	90	12	56	95	83
**500K**	Individual traits	1011	1595	1841	1994	1039	1601	1842	1991
	HIPO	974	1568	1830	1990	952	1552	1824	1984
	MTAG	1063	1607	1841	1994	1044	1591	1836	1988
	HIPO new	115	50	20	11	96	42	18	9
	MTAG new	93	31	9	4	69	22	7	2
		**Partial causal SNP overlap**				**Partial sample overlap**			
**10K**	Individual traits	0	0	1	3	0	0	1	1
	HIPO	0	0	1	3	0	0	0	1
	MTAG	0	0	1	4	0	0	1	1
	HIPO new	0	0	1	2	0	0	0	1
	MTAG new	0	0	1	2	0	0	0	1
**50K**	Individual traits	3	26	130	322	1	8	48	132
	HIPO	2	21	118	290	1	8	49	135
	MTAG	4	32	157	370	1	11	66	172
	HIPO new	2	13	56	105	1	5	25	57
	MTAG new	2	12	46	81	1	6	27	59
**100K**	Individual traits	26	187	629	1030	8	72	310	621
	HIPO	23	170	569	958	8	72	308	618
	MTAG	33	226	682	1074	12	95	377	712
	HIPO new	14	75	139	140	5	35	102	142
	MTAG new	13	62	98	86	6	37	100	132
**500K**	Individual traits	1033	1617	1875	2032	625	1355	1694	1851
	HIPO	963	1589	1867	2032	622	1347	1685	1848
	MTAG	1080	1628	1876	2033	718	1410	1708	1858
	HIPO new	139	66	28	14	139	99	44	24
	MTAG new	88	28	7	3	133	82	28	14

h^2_{max} is the largest heritability among the individual traits.
