# Supplementary material for: Heritability informed power optimization (HIPO) leads to enhanced detection of genetic associations across multiple traits
Source: PLoS Genet. 2018 Oct 5;14(10):e1007549. doi: 10.1371/journal.pgen.1007549 (PMC6192650; doi:10.1371/journal.pgen.1007549)
Supplement: S12 Table — (PDF) [file pgen.1007549.s012.pdf]

**S12 Table. Type I error rates for MTAG observed in datasets simulated under covariance structure estimated from studies of blood lipids with population stratification.** See S1 Table 1b for detailed settings. Summary-level association statistics are simulated for 4 traits using genetic and phenotypic covariance matrices estimated from Global Lipids Genetics Consortium (GLGC) data. The results for MTAG estimates of all 4 traits are listed. Reported are the average of genome-wide type I error rates across 100 simulations, under significance thresholds p<0.05, p<0.01 and p<0.001.

| N \ $h^2_{max}$ | p-value threshold | 0.1 | 0.2 | 0.35 | 0.5 | 0.1 | 0.2 | 0.35 | 0.5 |
|---|---|---|---|---|---|---|---|---|---|
| | | **MTAG 1** | | | | **MTAG 2** | | | |
| **10K** | p<0.05 | 0.051 | 0.05 | 0.05 | 0.05 | 0.05 | 0.05 | 0.05 | 0.05 |
| | p<0.01 | 0.01 | 0.01 | 0.01 | 0.01 | 0.01 | 0.01 | 0.01 | 0.01 |
| | p<0.001 | 0.0011 | 0.0011 | 0.0011 | 0.0011 | 0.001 | 0.001 | 0.001 | 0.001 |
| **50K** | p<0.05 | 0.05 | 0.05 | 0.05 | 0.05 | 0.05 | 0.05 | 0.05 | 0.05 |
| | p<0.01 | 0.01 | 0.01 | 0.01 | 0.01 | 0.01 | 0.01 | 0.01 | 0.01 |
| | p<0.001 | 0.001 | 0.001 | 0.001 | 0.001 | 0.001 | 0.001 | 0.001 | 0.001 |
| **100K** | p<0.05 | 0.05 | 0.05 | 0.05 | 0.05 | 0.05 | 0.05 | 0.05 | 0.05 |
| | p<0.01 | 0.01 | 0.01 | 0.01 | 0.01 | 0.01 | 0.01 | 0.01 | 0.01 |
| | p<0.001 | 0.001 | 0.001 | 0.0011 | 0.001 | 0.001 | 0.001 | 0.001 | 0.001 |
| **500K** | p<0.05 | 0.05 | 0.05 | 0.05 | 0.05 | 0.05 | 0.05 | 0.05 | 0.051 |
| | p<0.01 | 0.01 | 0.01 | 0.01 | 0.01 | 0.01 | 0.01 | 0.01 | 0.01 |
| | p<0.001 | 0.001 | 0.0011 | 0.001 | 0.001 | 0.001 | 0.001 | 0.0011 | 0.0011 |
| | | **MTAG 3** | | | | **MTAG 4** | | | |
| **10K** | p<0.05 | 0.05 | 0.05 | 0.05 | 0.05 | 0.051 | 0.05 | 0.05 | 0.05 |
| | p<0.01 | 0.01 | 0.01 | 0.01 | 0.01 | 0.01 | 0.01 | 0.01 | 0.01 |
| | p<0.001 | 0.001 | 0.001 | 0.001 | 0.001 | 0.001 | 0.001 | 0.0011 | 0.001 |
| **50K** | p<0.05 | 0.05 | 0.05 | 0.05 | 0.05 | 0.05 | 0.05 | 0.05 | 0.05 |
| | p<0.01 | 0.01 | 0.01 | 0.01 | 0.01 | 0.01 | 0.01 | 0.01 | 0.01 |
| | p<0.001 | 0.001 | 0.001 | 0.001 | 0.001 | 0.001 | 0.001 | 0.001 | 0.001 |
| **100K** | p<0.05 | 0.05 | 0.05 | 0.05 | 0.05 | 0.05 | 0.05 | 0.05 | 0.05 |
| | p<0.01 | 0.01 | 0.01 | 0.01 | 0.01 | 0.01 | 0.01 | 0.01 | 0.01 |
| | p<0.001 | 0.001 | 0.0011 | 0.001 | 0.0011 | 0.001 | 0.001 | 0.0011 | 0.001 |
| **500K** | p<0.05 | 0.05 | 0.05 | 0.05 | 0.05 | 0.05 | 0.05 | 0.05 | 0.05 |
| | p<0.01 | 0.01 | 0.01 | 0.01 | 0.01 | 0.01 | 0.01 | 0.01 | 0.01 |
| | p<0.001 | 0.001 | 0.001 | 0.0011 | 0.0011 | 0.001 | 0.0011 | 0.001 | 0.001 |

$h^2_{max}$ is the largest heritability among the individual traits. MTAG 1/2/3/4: MTAG estimate for trait 1/2/3/4.
