# Supplementary material for: Heritability informed power optimization (HIPO) leads to enhanced detection of genetic associations across multiple traits
Source: PLoS Genet. 2018 Oct 5;14(10):e1007549. doi: 10.1371/journal.pgen.1007549 (PMC6192650; doi:10.1371/journal.pgen.1007549)
Supplement: S13 Table — (PDF) [file pgen.1007549.s013.pdf]

S13 Table. Type I error rates for HIPO observed in simulated unbalanced case-control datasets. Data are simulated using UK Biobank individual level genotypes of ~50K randomly selected subjects. Three genetically correlated binary phenotypes are simulated using logistic regression with the same genetic covariance matrix as scenario 2a in S1 Table (see Section E.1 of S1 Appendix for details). Reported are the average of genome-wide type I error rates across 100 simulations, under significance thresholds p<0.05, p<0.01 and p<0.001.

	**p-value threshold**	**Prevalence 0.05**	**Prevalence 0.1**	**Prevalence 0.2**
**HIPO-D1**	p<0.05	0.051	0.051	0.051
	p<0.01	0.01	0.01	0.01
	p<0.001	0.0011	0.001	0.0011
**HIPO-D2**	p<0.05	0.05	0.051	0.051
	p<0.01	0.01	0.01	0.01
	p<0.001	0.001	0.0011	0.001
**HIPO-D3**	p<0.05	0.049	0.05	0.05
	p<0.01	0.01	0.01	0.01
	p<0.001	0.001	0.001	0.001
