# Supplementary material for: Heritability informed power optimization (HIPO) leads to enhanced detection of genetic associations across multiple traits
Source: PLoS Genet. 2018 Oct 5;14(10):e1007549. doi: 10.1371/journal.pgen.1007549 (PMC6192650; doi:10.1371/journal.pgen.1007549)
Supplement: S14 Table — Numbers in the parentheses are the heritability estimated using LD score regression. (PDF) [file pgen.1007549.s014.pdf]

S14 Table. Weights associated with individual blood lipid traits and average non-centrality parameters for each HIPO component. Numbers in the parentheses are the heritability estimated using LD score regression.

	HIPO-D1	HIPO-D2	HIPO-D3	HIPO-D4
**LDL (0.146)**	0.147	0.206	0.820	-2.156
**HDL (0.168)**	-0.618	-0.017	-0.611	-1.032
**TG (0.156)**	0.591	0.228	-0.822	-0.778
**TC (0.159)**	-0.469	0.765	-0.343	2.387
**Average NCP**	0.213	0.175	0.080	0.026

TG: triglycerides; TC: total cholesterol.
