# Supplementary material for: Heritability informed power optimization (HIPO) leads to enhanced detection of genetic associations across multiple traits
Source: PLoS Genet. 2018 Oct 5;14(10):e1007549. doi: 10.1371/journal.pgen.1007549 (PMC6192650; doi:10.1371/journal.pgen.1007549)
Supplement: S15 Table — Numbers in the parentheses are the heritability estimated using LD score regression. (PDF) [file pgen.1007549.s015.pdf]

**S15 Table. Weights associated with individual psychiatric diseases and average non-centrality parameters for each HIPO component.** Numbers in the parentheses are the heritability estimated using LD score regression.

| | HIPO-D1 | HIPO-D2 | HIPO-D3 | HIPO-D4 | HIPO-D5 |
|---|---|---|---|---|---|
| **ASD (1.551)** | 0.082 | 0.917 | -0.177 | -0.123 | -0.178 |
| **ADHD (0.685)** | 0.025 | -0.141 | -0.048 | 0.058 | -0.676 |
| **BIP (1.962)** | 0.542 | -0.231 | -0.551 | -0.589 | 0.054 |
| **MDD (0.623)** | 0.257 | -0.004 | -0.705 | 0.928 | 0.144 |
| **SCZ (2.238)** | 0.968 | 0.075 | 0.699 | 0.231 | -0.012 |
| **Average NCP** | 4.651 | 1.493 | 0.899 | 0.566 | 0.275 |

Heritability reported in the log-OR scale to make them comparable to the average NCP. ASD: autism spectrum disorder; BIP: bipolar disorder; MDD: major depressive disorder; SCZ: schizophrenia.
