# Supplementary material for: Heritability informed power optimization (HIPO) leads to enhanced detection of genetic associations across multiple traits
Source: PLoS Genet. 2018 Oct 5;14(10):e1007549. doi: 10.1371/journal.pgen.1007549 (PMC6192650; doi:10.1371/journal.pgen.1007549)
Supplement: S16 Table — Numbers in the parentheses are the heritability estimated using LD score regression. (PDF) [file pgen.1007549.s016.pdf]

S16 Table. Weights associated with individual social science traits and average non-centrality parameters for each HIPO component. Numbers in the parentheses are the heritability estimated using LD score regression.

	HIPO-D1	HIPO-D2	HIPO-D3
**DS (0.060)**	-0.247	-0.605	0.691
**NEU (0.092)**	-0.607	0.796	-0.010
**SWB (0.036)**	0.588	0.640	0.855
**Average NCP**	0.101	0.031	0.018

DS: depressive symptoms; NEU: neuroticism; SWB: subjective well-being.
