# Supplementary material for: Heritability informed power optimization (HIPO) leads to enhanced detection of genetic associations across multiple traits
Source: PLoS Genet. 2018 Oct 5;14(10):e1007549. doi: 10.1371/journal.pgen.1007549 (PMC6192650; doi:10.1371/journal.pgen.1007549)
Supplement: S17 Table — Only HIPO-D1 and HIPO-D2 are considered. (PDF) [file pgen.1007549.s017.pdf]

S17 Table. Novel loci for blood lipids identified by HIPO and MTAG. Only HIPO-D1 and HIPO-D2 are considered.

SNP	CHR	P_{LDL}	P_{HDL}	P_{TG}	P_{TC}	$P_{HIPO\text{-}D1}$	$P_{HIPO\text{-}D2}$	$P_{LDL,MTAG}$	$P_{HDL,MTAG}$	$P_{TG,MTAG}$	$P_{TC,MTAG}$
**Common (15)**											
rs17199964	4	3.11e-01	9.19e-08	1.27e-01	9.85e-04	3.03e-08	2.43e-02	3.50e-02	8.88e-09	1.55e-02	3.97e-04
rs6855363	4	6.44e-01	3.20e-07	3.18e-04	5.89e-01	2.27e-08	5.44e-01	7.97e-01	2.74e-08	8.66e-06	4.58e-01
rs176813	4	2.62e-05	1.75e-01	4.23e-04	5.68e-08	5.62e-01	8.10e-09	4.32e-07	3.29e-01	1.54e-03	1.34e-08
rs2268719	6	7.52e-07	3.08e-01	4.14e-02	6.66e-08	9.39e-01	2.13e-08	1.54e-08	6.20e-01	9.70e-03	5.15e-08
rs11987974	8	7.66e-01	3.79e-06	1.85e-06	7.16e-01	3.91e-09	3.32e-01	8.53e-01	4.10e-08	8.91e-08	5.96e-01
rs661171	11	9.06e-03	9.84e-07	1.77e-01	2.70e-06	2.58e-08	2.23e-04	1.95e-04	3.56e-08	4.45e-02	7.18e-07
rs10832027	11	2.53e-02	1.52e-07	5.73e-07	1.87e-02	3.88e-12	3.16e-01	3.76e-02	4.90e-10	2.86e-08	8.21e-03
rs7938117	11	3.69e-01	2.05e-07	9.49e-06	4.01e-02	3.35e-11	5.30e-01	1.78e-01	6.52e-10	5.24e-07	2.43e-02
rs2384034	12	9.47e-03	3.61e-07	3.18e-02	5.00e-05	4.39e-09	2.51e-03	1.00e-03	1.24e-08	4.48e-03	1.35e-05
rs895953	12	7.23e-01	1.45e-06	3.98e-07	5.43e-01	2.84e-10	3.86e-01	9.60e-01	5.31e-09	2.08e-08	4.57e-01
rs721772	15	5.17e-01	2.26e-07	4.30e-05	7.10e-01	4.25e-09	3.75e-01	6.69e-01	8.65e-09	9.57e-07	5.79e-01
rs11079810	17	1.99e-02	1.71e-07	2.23e-04	9.51e-03	4.30e-10	1.32e-01	2.65e-02	5.56e-09	1.06e-05	3.46e-03
rs4805755	19	9.34e-01	5.58e-08	4.83e-03	9.85e-02	7.71e-09	6.28e-01	5.56e-01	4.03e-09	1.91e-04	6.20e-02
rs10408163	19	1.47e-01	9.99e-07	3.20e-07	2.70e-01	8.87e-09	1.65e-02	9.36e-02	3.84e-08	4.52e-09	3.42e-01
rs6059932	20	1.30e-01	5.73e-07	6.33e-05	6.33e-02	1.27e-09	4.76e-01	1.49e-01	1.51e-08	3.08e-06	3.30e-02
**HIPO\MTAG (9)**											
rs4850047	2	2.87e-03	1.15e-04	8.58e-04	2.14e-06	1.13e-09	7.82e-04	2.88e-05	1.33e-07	7.81e-04	8.99e-07
rs2249105	2	8.72e-02	6.35e-06	1.89e-06	4.66e-01	1.33e-08	7.92e-01	4.14e-01	4.14e-07	8.36e-08	3.00e-01
rs2062432	3	9.78e-01	3.88e-06	2.44e-03	6.75e-02	4.30e-08	5.52e-01	3.95e-01	7.91e-08	2.68e-04	4.98e-02
rs4683438	3	5.70e-05	6.85e-01	3.77e-04	2.87e-07	5.41e-01	2.99e-08	6.24e-07	6.28e-01	2.16e-04	1.54e-07
rs10054063	5	7.00e-01	6.13e-04	7.72e-07	2.67e-01	3.69e-08	7.80e-01	4.30e-01	1.38e-06	4.23e-07	2.14e-01
rs740746	10	4.32e-01	1.09e-06	1.61e-03	1.07e-01	4.21e-08	5.74e-01	3.30e-01	9.09e-08	8.98e-05	6.32e-02
rs1565228	11	2.46e-01	3.50e-05	3.44e-06	7.19e-02	2.56e-09	6.44e-01	1.57e-01	1.41e-07	7.75e-07	5.13e-02
rs7939352	11	2.79e-06	9.75e-01	5.47e-03	2.48e-07	9.57e-01	3.47e-08	1.32e-07	9.91e-01	4.25e-03	8.79e-08
rs11048456	12	8.65e-02	2.93e-07	1.59e-03	5.74e-02	1.75e-08	3.28e-01	1.37e-01	5.30e-08	6.49e-05	2.56e-02
**MTAG\HIPO (10)**											
rs2301453	1	1.07e-03	1.03e-05	1.69e-06	5.47e-03	1.88e-06	7.44e-05	4.83e-04	1.72e-06	2.08e-08	6.78e-03
rs10900522	1	8.34e-01	8.80e-08	3.76e-03	5.75e-01	1.24e-07	7.04e-01	8.29e-01	4.96e-08	8.30e-05	4.06e-01
rs6541229	1	1.72e-04	5.59e-05	6.71e-07	5.46e-04	1.08e-05	4.00e-06	4.26e-05	1.10e-05	1.05e-08	6.16e-04
rs10185855	2	2.50e-07	4.24e-01	4.44e-01	1.77e-07	4.91e-01	1.85e-07	1.47e-08	9.49e-01	1.62e-01	1.12e-07
rs4897361	6	2.95e-03	1.65e-03	3.04e-07	2.28e-04	1.92e-04	3.52e-06	1.22e-04	4.90e-04	1.42e-08	2.74e-04
rs2845885	11	2.65e-01	4.28e-06	2.07e-06	1.66e-01	2.33e-07	1.42e-02	8.28e-02	7.27e-07	3.17e-08	2.33e-01
rs12423664	12	1.15e-03	3.30e-02	1.44e-07	1.02e-04	1.65e-03	1.15e-06	7.58e-05	6.30e-03	3.73e-08	8.30e-05
rs10876041	12	2.17e-06	1.70e-01	3.03e-01	8.73e-08	4.80e-02	1.56e-07	1.11e-07	9.00e-02	3.04e-01	2.50e-08
rs2854322	17	1.16e-05	2.45e-01	1.92e-01	5.11e-08	7.48e-02	1.09e-07	1.46e-07	1.20e-01	1.74e-01	1.89e-08
rs11652146	17	1.74e-01	9.64e-08	1.40e-02	5.81e-01	2.18e-07	5.98e-01	5.04e-01	2.83e-08	4.77e-04	4.66e-01

Independent SNPs were identified through LD-pruning with r^2 threshold of 0.1 and pruned SNPs were assumed to represent independent loci if they are >0.5Mb apart. Loci are considered novel if they are not identified at genome-wide significance level through analysis of individual traits.

Common: identified by both HIPO and MTAG; HIPO\MTAG: identified by HIPO but not MTAG; MTAG\HIPO: identified by MTAG but not HIPO.
