# Supplementary material for: Heritability informed power optimization (HIPO) leads to enhanced detection of genetic associations across multiple traits
Source: PLoS Genet. 2018 Oct 5;14(10):e1007549. doi: 10.1371/journal.pgen.1007549 (PMC6192650; doi:10.1371/journal.pgen.1007549)
Supplement: S18 Table — Only HIPO-D1 is considered. (PDF) [file pgen.1007549.s018.pdf]

S18 Table. Novel loci for social science traits identified by HIPO and MTAG. Only HIPO-D1 is considered.

SNP	CHR	P_{DS}	P_{NEU}	P_{SWB}	$P_{HIPO\text{-}D1}$	$P_{DS,MTAG}$	$P_{NEU,MTAG}$	$P_{SWB,MTAG}$
**Common (12)**								
rs2874367	1	6.33e-05	6.33e-05	6.33e-05	1.38e-08	2.22e-08	4.09e-07	4.65e-08
rs11100449	4	1.47e-05	6.33e-05	6.33e-05	8.02e-09	9.75e-09	3.45e-07	3.01e-08
rs10475748	5	1.47e-05	5.73e-07	1.96e-02	1.90e-08	3.53e-08	5.09e-08	2.48e-06
rs6919210	6	1.15e-03	5.73e-07	1.77e-04	1.88e-09	1.28e-08	7.64e-09	3.66e-08
rs6569095	6	8.58e-04	2.14e-05	1.47e-05	5.94e-09	2.25e-08	1.06e-07	1.54e-08
rs210899	6	1.10e-01	2.03e-06	6.33e-05	3.61e-08	6.70e-07	4.35e-08	1.85e-07
rs2396726	7	1.77e-04	3.06e-06	8.58e-04	1.04e-08	3.05e-08	6.29e-08	2.21e-07
rs12701427*	7	1.15e-03	1.47e-05	6.33e-05	1.28e-08	5.02e-08	1.30e-07	5.78e-08
rs9584850	13	6.33e-05	1.52e-07	8.58e-04	6.50e-10	2.66e-09	2.90e-09	4.48e-08
rs11644362	16	2.70e-03	2.46e-04	3.06e-06	3.73e-08	1.20e-07	1.04e-06	2.22e-08
rs1261093	18	8.58e-04	9.64e-08	2.70e-03	3.92e-09	2.65e-08	5.16e-09	3.30e-07
rs7239568	18	5.96e-03	2.14e-05	9.64e-08	8.17e-10	6.91e-09	2.60e-08	3.81e-10
**MTAG\HIPO (2)**								
rs1075737	7	4.65e-04	2.70e-03	2.03e-06	1.59e-07	2.07e-07	1.17e-05	3.70e-08
rs11610143	12	1.64e-02	1.77e-04	2.03e-06	5.54e-08	3.05e-07	8.55e-07	2.83e-08

Independent SNPs were identified through LD-pruning with r^2 threshold of 0.1 and pruned SNPs were assumed to represent independent loci if they are >0.5Mb apart. Loci are considered novel if they are not identified at genome-wide significance level through analysis of individual traits.

Common: identified by both HIPO and MTAG; HIPO\MTAG: identified by HIPO but not MTAG; MTAG\HIPO: identified by MTAG but not HIPO.

*MTAG detects locus rs12701427 by a nearby SNP rs588123 (~12Kb away), although the p-values for rs12701427 is slightly above 5e-8.
