# Supplementary material for: Heritability informed power optimization (HIPO) leads to enhanced detection of genetic associations across multiple traits
Source: PLoS Genet. 2018 Oct 5;14(10):e1007549. doi: 10.1371/journal.pgen.1007549 (PMC6192650; doi:10.1371/journal.pgen.1007549)
Supplement: S1 Appendix — (PDF) [file pgen.1007549.s019.pdf]

S1 Appendix

A Estimation of Σ_g and $\Sigma_{\hat{\beta}}$

In this appendix we show how to use LD score regression[1, 2] to estimate the genetic covariance Σ_g and covariance matrix of the GWAS estimates $\Sigma_{\hat{\beta}}$. The same technique was used in MTAG[3], but here we describe it in more detail. Assume that the genotype and phenotype data are both standardized to have mean 0 and variance 1. The diagonal elements of Σ_g are the values of heritability, and therefore can be estimated by single trait LD score regression:

$$E[\chi^2_{jk} \mid l_j] = \frac{N_{jk} h_k^2}{M} l_j + 1,$$

where N_{jk} is the sample size for SNP j and the kth trait, h_k^2 is the heritability and l_j is the LD score. The diagonal elements of $\Sigma_{\hat{\beta}}$ is simply $\frac{1}{N_{jk}}$.

To estimate the cross-trait parameters, we write LD score regression in a different form. Denote by $\beta_{jk}^{(J)}$ the joint effect size, β_{jk} the marginal effect size and $\hat{\beta}_{jk}$ the GWAS estimate for SNP j and the kth trait. Denote by h_{kl} the

genetic covariance of the kth and lth trait. We have

$$\begin{aligned}
E[\hat{\beta}_{jk}\hat{\beta}_{jl} \mid l_j] &= E[(\hat{\beta}_{jk} - \beta_{jk} + \beta_{jk})(\hat{\beta}_{jl} - \beta_{jl} + \beta_{jl}) \mid l_j] \\
&= E[(\hat{\beta}_{jk} - \beta_{jk})(\hat{\beta}_{jl} - \beta_{jl}) \mid l_j] + E[\beta_{jk}\beta_{jl} \mid l_j] \\
&+ E[(\hat{\beta}_{jl} - \beta_{jl})\beta_{jk} \mid l_j] + E[(\hat{\beta}_{jk} - \beta_{jk})\beta_{jl} \mid l_j] \\
&= cov(\hat{\beta}_{jk}, \hat{\beta}_{jl}) + E\Big[\Big(\sum_{j'=1}^{M} r_{jj'}\beta_{jk}^{(J)}\Big)\Big(\sum_{j'=1}^{M} r_{jj'}\beta_{jl}^{(J)}\Big) \mid l_j\Big] \\
&= cov(\hat{\beta}_{jk}, \hat{\beta}_{jl}) + \frac{h_{kl}}{M} l_j.
\end{aligned}$$

Therefore

$$E[z_{jk}z_{jl} \mid l_j] = \sqrt{N_{jk}N_{jl}} cov(\hat{\beta}_{jk}, \hat{\beta}_{jl}) + \frac{\sqrt{N_{jk}N_{jl}} h_{kl}}{M} l_j,$$

of which the slope and intercept can be used to estimate the off-diagonal elements of Σ_g and $\Sigma_{\hat{\beta}}$ respectively.

B HIPO Eigendecomposition

Here we explain the details on solving the optimization problem

$$max_{\boldsymbol{c}} \boldsymbol{c}^T \hat{\Sigma}_g \boldsymbol{c} \quad \text{subject to } \boldsymbol{c}^T \hat{\Sigma}_{\hat{\beta}} \boldsymbol{c} = 1.$$

Similar optimization problems have been dealt with in previous papers[4, 5]. By Cholesky decomposition $\hat{\Sigma}_{\hat{\beta}} = R^T R$ and transformation $\boldsymbol{\phi} = R\boldsymbol{c}$, it is equivalent to the problem

$$max_{\boldsymbol{\phi}} \boldsymbol{\phi}^T R^{-T} \hat{\Sigma}_g R^{-1} \boldsymbol{\phi} \quad \text{subject to } \boldsymbol{\phi}^T \boldsymbol{\phi} = 1.$$

Using Lagrange multiplier method, the problem above is transformed into maximization of

$$\boldsymbol{\phi}^T R^{-T} \hat{\Sigma}_g R^{-1} \boldsymbol{\phi} - \lambda(\boldsymbol{\phi}^T \boldsymbol{\phi} - 1).$$

Differentiate with respect to $\boldsymbol{\phi}$ gives

$$R^{-T} \hat{\Sigma}_g R^{-1} \boldsymbol{\phi} = \lambda \boldsymbol{\phi},$$

i.e. $\boldsymbol{\phi}$ is an eigenvector of $R^{-T} \hat{\Sigma}_g R^{-1}$ and $\boldsymbol{\phi}^T R^{-T} \hat{\Sigma}_g R^{-1} \boldsymbol{\phi} = \lambda$. Therefore $\boldsymbol{\phi}$ is the eigenvector corresponding to the largest eigenvalue of $R^{-T} \hat{\Sigma}_g R^{-1}$, denoted by $\boldsymbol{\phi}_1$ and λ_1.

Subsequent weights $\boldsymbol{\phi}_k$ $(k = 2, ..., K)$ are defined similarly but required to be orthogonal to previously defined $\boldsymbol{\phi}$'s. Eigendecompostition of $R^{-T} \hat{\Sigma}_g R^{-1}$ gives eigenvalues $\lambda_1 \geq ... \geq \lambda_K$ and eigenvectors $\boldsymbol{\phi}_1, ..., \boldsymbol{\phi}_K$. The HIPO component $\boldsymbol{c}_k$ is given by $\boldsymbol{c}_k = R^{-1} \boldsymbol{\phi}_k$.

Denote by $E[\delta_k]$ the average NCP corresponding to $\boldsymbol{c}_k$, we have

$$E[\delta_k] = A \frac{\boldsymbol{c}_k^T \Sigma_g \boldsymbol{c}_k}{\boldsymbol{c}_k^T \Sigma_{\hat{\beta}} \boldsymbol{c}_k} = A \boldsymbol{c}_k^T \Sigma_g \boldsymbol{c}_k = A \lambda_k,$$

where the constant $A = \frac{E[l]}{M}$. This shows that the eigenvalues are proportional to the average non-centrality parameter. Furthermore,

$$cov(\boldsymbol{c}_k^T \hat{\boldsymbol{\beta}}, \boldsymbol{c}_l^T \hat{\boldsymbol{\beta}}) = \boldsymbol{c}_k^T \hat{\Sigma}_{\hat{\beta}} \boldsymbol{c}_l = \boldsymbol{\phi}_k^T \boldsymbol{\phi}_l = 0,$$

Therefore $\boldsymbol{c}_k^T \hat{\boldsymbol{\beta}}$ are uncorrelated and in decreasing order of $E[\delta]$.

C Discussion of the case where sample size is different across SNPs within the same study

We only derive HIPO assuming the sample size is the same across all SNPs within the same study. This is approximately true in most studies, and we also filtered out the SNPs with sample size less than 0.67 times the 90th percentile of the sample size to make it homogeneous. However, in some cases, sample size can be different for different SNPs due to missingness and difference in chip design. Here we show that our method is still valid in this case.

In the main paper, we maximize the quantity

$$E[\delta] = \frac{E[(\boldsymbol{c}^T\boldsymbol{\beta})^2]}{var(\boldsymbol{c}^T\hat{\boldsymbol{\beta}})}$$

It still holds that $E[\boldsymbol{c}^T\boldsymbol{\beta}] = \frac{E[l]}{M}\boldsymbol{c}^T\Sigma_g\boldsymbol{c}$. The denominator is slightly different if the sample size varies across SNPs:

$$cov(\hat{\beta}_{jk}, \hat{\beta}_{j,l}) = \frac{N_{jkl}}{N_{jk}N_{jl}}cov(y_k, y_l) = \frac{1}{\sqrt{N_{jk}N_{jl}}}\frac{N_{jkl}}{\sqrt{N_{jk}N_{jl}}}cov(y_k, y_l).$$

The ratio $\frac{N_{jkl}}{\sqrt{N_{jk}N_{jl}}}$ captures the proportion of overlapping subjects and should be nearly constant if the sample is homogeneous. The SNP-specific sample size N_{jk} can be viewed as random variable that has mean aN_k, where N_k is the total sample size and $0 \leq a \leq 1$ is the expected proportion of non-missing subjects that is the same for all studies, therefore

$$cov(\hat{\beta}_{jk}, \hat{\beta}_{jl}) = \frac{N_{kl}}{aN_kN_l}cov(y_k, y_l).$$

Therefore, even if sample size varies across SNPs, the average NCP $E[\delta]$ is the same as the average NCP if all SNPs are genotyped on all subjects, up to

multiplication of a constant. The maximization procedure still holds and gives the same answer.

D Justification of the simulation procedure

Here we derive the joint distribution of the simulation error term $\tilde{\boldsymbol{e}}$ and show the error term generated based on the 1000 Genomes reference panel has the desired distribution.

Suppose we pick two SNPs j and j' and two phenotypes y_k and y_l. For simplicity, we assume that the sample size is the same for all SNPs within the same study. The GWAS parameter estimates can be decomposed into the sum of the true effect size and the error

$$\hat{\beta}_{jk} = \beta_{jk} + e_{jk},\ \hat{\beta}_{j'l} = \beta_{j'l} + e_{j'l}.$$

The covariance of the error terms is

$$\begin{aligned} cov(e_{jk}, e_{j'l}) &= cov\Big(\frac{1}{N_k} X_{jk}^T Y_k, \frac{1}{N_l} X_{j'l}^T Y_l\Big) & (1) \\ &= \frac{1}{N_k N_l} X_{jk}^T cov(Y_k, Y_l) X_{j'l} & (2) \\ &\approx \frac{N_{kl}}{N_k N_l} cov(y_k, y_l) r_{jj'}, & (3) \end{aligned}$$

where N_k and N_l are the sample sizes and N_{kl} is the sample overlap. From (2) to (3), we utilize that fact that $cov(Y_k, Y_l)$ is a N_k by N_l matrix that has the

form

$$\begin{pmatrix} cov(y_k, y_l) & 0 & 0 & ... & 0 \\ 0 & cov(y_k, y_l) & 0 & ... & 0 \\ 0 & 0 & cov(y_k, y_l) & ... & 0 \\ ... & ... & ... & ... & ... \\ 0 & 0 & 0 & ... & 0 \end{pmatrix}.$$

The 1st to N_{kl}-th diagonal elements are the phenotypic covariance $cov(y_k, y_l)$. The rest of the elements are 0.

Therefore, the covariance matrix of the GWAS error term is $R \otimes \Sigma_e$, where R is the matrix of LD coefficients and Σ_e is a K by K matrix of which the (k, l) element is $\frac{N_{kl}}{N_k N_l} cov(y_k, y_l)$.

When the traits are independent of the genotype, i.e. $\beta_{jk} = \beta_{j'l} = 0$, we have

$$cov(\hat{\beta}_{jk}, \hat{\beta}_{j'l}) = cov(e_{jk}, e_{j'l}) \approx \frac{N_{kl}}{N_k N_l} cov(y_k, y_l) r_{jj'}.$$

The quantity $\frac{N_{kl}}{\sqrt{N_k N_l}}$ is approximately invariant to proportional scaling of sample sizes if the samples are homogeneous. Let n_k, n_l and n_{kl} are the sample sizes and sample overlap in 1000 Genomes reference panel. Thus,

$$cov(\hat{\beta}_{jk,1000G}, \hat{\beta}_{j'l,1000G}) = \frac{1}{\sqrt{n_k n_l}} \frac{n_{kl}}{\sqrt{n_k n_l}} cov(y_k, y_l) r_{jj'} \approx \frac{1}{\sqrt{n_k n_l}} \frac{N_{kl}}{\sqrt{N_k N_l}} cov(y_k, y_l) r_{jj'}.$$

Rescaling the 1000 Genomes estimates give the desired distribution, since

$$cov\Big(\frac{\sqrt{n_k}}{\sqrt{N_k}} \hat{\beta}_{jk,1000G}, \frac{\sqrt{n_l}}{\sqrt{N_l}} \hat{\beta}_{j'l,1000G}\Big) \approx \frac{N_{kl}}{N_k N_l} cov(y_k, y_l) r_{jj'}.$$

Therefore, we can simulate the error terms by generating null phenotypes and conduct one SNP at a time association analysis on the 1000 Genomes reference panel.

E Additional simulation settings

E.1 Unbalanced case-control studies

We also conduct simulations using UK Biobank individual-level genotype data to study the type I error of HIPO under unbalanced case-control designs. We try to randomly draw fifty thousand subjects and 48,756 are kept by software QCTOOL from Oxford (`http://www.well.ox.ac.uk/~gav/qctool_v2/`). We only use ∼1.07 million SNPs that appear in HapMap3. Three traits are simulated using logistic model

$$logit(p_k) = \alpha + \sum_{\{j\ is\ causal\}} \beta_{jk}^{(J)} X_j, \quad k = 1, 2, 3$$

where p_k is the probability of being a case for trait k and X_j is the genotype of SNP j. The intercept α is assumed to be the same for all 3 traits. We randomly select ∼10.7K SNPs as causal variants that are shared by all traits and the effect sizes of the causal SNPs are simulated as $\boldsymbol{\beta}_j^{(J)} \sim N(\mathbf{0}, \Sigma_g/10.7K)$. Here Σ_g is the genetic covariance in scenario 2a (**S1 Table**) with $h^2_{max} = 0.5$. Three traits are generated from independent Bernoulli distributions with probability p_1, p_2 and p_3, respectively. We vary the intercept $\alpha = log(0.05/(1-0.05)), log(0.1/(1-0.1)), log(0.2/(1-0.2))$ to mimic diseases of prevalence 5%, 10% and 20%. Standard association analysis is performed with one SNP at a time logistic regression adjusting for 10 genetic PCs. The simulation is repeated 100 times.

E.2 Exploring the number of dominant HIPO components and underlying genetic mechanisms

HIPO can project multivariate phenotypes onto genetically independent directions. We hence conjecture that in some cases, the number of dominant HIPO components (with average NCP far larger than the following components) should be equal to the number of underlying genetic mechanisms. We conduct a simple simulation study to explore this property.

We simulate 4 traits of which the genetic components come from two independent mechanisms. Twenty-five thousand subjects are randomly selected from UK Biobank and 24,949 are kept by the software QCTOOL. Same as last subsection, we only use ∼1.07 million SNPs that appear in HapMap3. Four phenotypes $\boldsymbol{y} = (y_1, y_2, y_3, y_4)^T$ are generated by simulating the genetic component $\boldsymbol{g} = (g_1, g_2, g_3, g_4)^T$ and environmental component $\boldsymbol{e}$ separately and summing them up. We first randomly select ∼10.7K SNPs as causal variants shared by all 4 traits. We then simulate g_1 and g_2 independently using linear model

$$g_k = \sum_{\{j \ is \ causal\}} \beta_{jk}^{(J)} X_j, \quad k = 1, 2.$$

Here $\beta_{j1}^{(J)}, \beta_{j2}^{(J)} \sim N(0, \frac{0.3}{10.7K})$. The third and fourth components are defined as linear combinations of the first and second: $g_3 = 0.6g_1 + 0.8g_2, g_4 = 0.8g_1 + 0.6g_2$. The environmental component $\boldsymbol{e}$ is generated from multivariate normal

distribution $N(\mathbf{0}, D)$, where

$$D = 0.7 * \begin{pmatrix} 1 & 0.5 & 0.5 & 0.5 \\ 0.5 & 1 & 0.5 & 0.5 \\ 0.5 & 0.5 & 1 & 0.5 \\ 0.5 & 0.5 & 0.5 & 1 \end{pmatrix}.$$

Therefore, all four traits have heritability 0.3 and are generated by two independent genetic mechanisms. Standard association analysis is performed with one SNP at a time linear regression, adjusting for 10 genetic PCs. The simulation is repeated 100 times.

Simulation results: The mean (standard deviation) of eigenvalues corresponding to HIPO-D1 to HIPO-D4 over 100 simulations are 0.479 (0.031), 0.359 (0.026), 0.015 (0.018) and -0.018 (0.017). The number of dominating HIPO-components (HIPO-D1 and HIPO-D2) is the same as the number of independent genetic mechanisms. This shows that HIPO can provide valuable insights to the sources of genetic signal in some scenarios.

F LD clumping procedure

Here we provide the details of our LD clumping algorithm. To ensure that one locus is always marked by the same SNP across all traits and HIPO components, we first identify the total set of independent loci detected by any individual trait or HIPO component and then "assign" these loci to traits or HIPO components.

We follow the steps below:

1. Compute the minimum p-values across all traits and all HIPO components we consider, i.e.

$$p_{min} = min\{p_{trait,1}, p_{trait,2}, ..., p_{trait,K}, p_{HIPO-D1}, ..., p_{HIPO-DK'}\},$$

where K is the number of traits and K' is the number of HIPO components of interest, which may be smaller than K.

2. Do LD clumping based on p_{min} under threshold $r^2 < 0.1$ and different loci required to be >0.5Mb apart.

3. "Assign" loci to an individual trait or HIPO component if either the lead SNP of the locus or a SNP in its clump satisfy $p < 5 \times 10^{-8}$ with the trait or HIPO component.

To incorporate the comparison between HIPO and MTAG without changing the previous results, we conduct LD clumping for the minimum p-value among all MTAG estimates, separately from individual traits or HIPO components. We label a locus as an "old" locus found by individual traits or HIPO if it's within 0.5Mb or satisfy $r^2 > 0.5$ with previously identified loci.

References

[1] Brendan K Bulik-Sullivan, Po-Ru Loh, Hilary K Finucane, Stephan Ripke, Jian Yang, Schizophrenia Working Group of the Psychiatric Genomics Consortium, Nick Patterson, Mark J Daly, Alkes L Price, and Benjamin M

Neale. LD score regression distinguishes confounding from polygenicity in genome-wide association studies. *Nat Genet*, 47(3):291–295, 03 2015.

[2] Brendan Bulik-Sullivan, Hilary K Finucane, Verneri Anttila, Alexander Gusev, Felix R Day, Po-Ru Loh, ReproGen Consortium, Psychiatric Genomics Consortium, Genetic Consortium for Anorexia Nervosa of the Wellcome Trust Case Control Consortium 3, Laramie Duncan, John R B Perry, Nick Patterson, Elise B Robinson, Mark J Daly, Alkes L Price, and Benjamin M Neale. An atlas of genetic correlations across human diseases and traits. *Nat Genet*, 47(11):1236–1241, 11 2015.

[3] Patrick Turley, Raymond K Walters, Omeed Maghzian, Aysu Okbay, James J Lee, Mark Alan Fontana, Tuan Anh Nguyen-Viet, Robbee Wedow, Meghan Zacher, Nicholas A Furlotte, et al. Mtag: multi-trait analysis of gwas. *bioRxiv*, page 118810, 2017.

[4] Lambertus Klei, Diana Luca, B Devlin, and Kathryn Roeder. Pleiotropy and principal components of heritability combine to increase power for association analysis. *Genetic Epidemiology*, 32(1):9–19, 2008.

[5] Jin J Zhou, Michael H Cho, Christoph Lange, Sharon Lutz, Edwin K Silverman, and Nan M Laird. Integrating multiple correlated phenotypes for genetic association analysis by maximizing heritability. *Human heredity*, 79(2):93—104, 2015.
