# Supplementary material for: Heritability informed power optimization (HIPO) leads to enhanced detection of genetic associations across multiple traits
Source: PLoS Genet. 2018 Oct 5;14(10):e1007549. doi: 10.1371/journal.pgen.1007549 (PMC6192650; doi:10.1371/journal.pgen.1007549)

S3 Fig. QQ plot of HIPO-D1 and the most heritable trait observed in datasets simulated under covariance structure of psychiatric diseases WITHOUT population stratification effects. We simulate 3 traits using genetic and phenotypic covariance matrices of autism spectrum disorder, bipolar disorder and schizophrenia estimated from Psychiatric Genomics Consortium (PGC) data. The dots represent the average quantiles across 100 simulations and the shade represents the range between 5% lower limit and 95% upper limit.

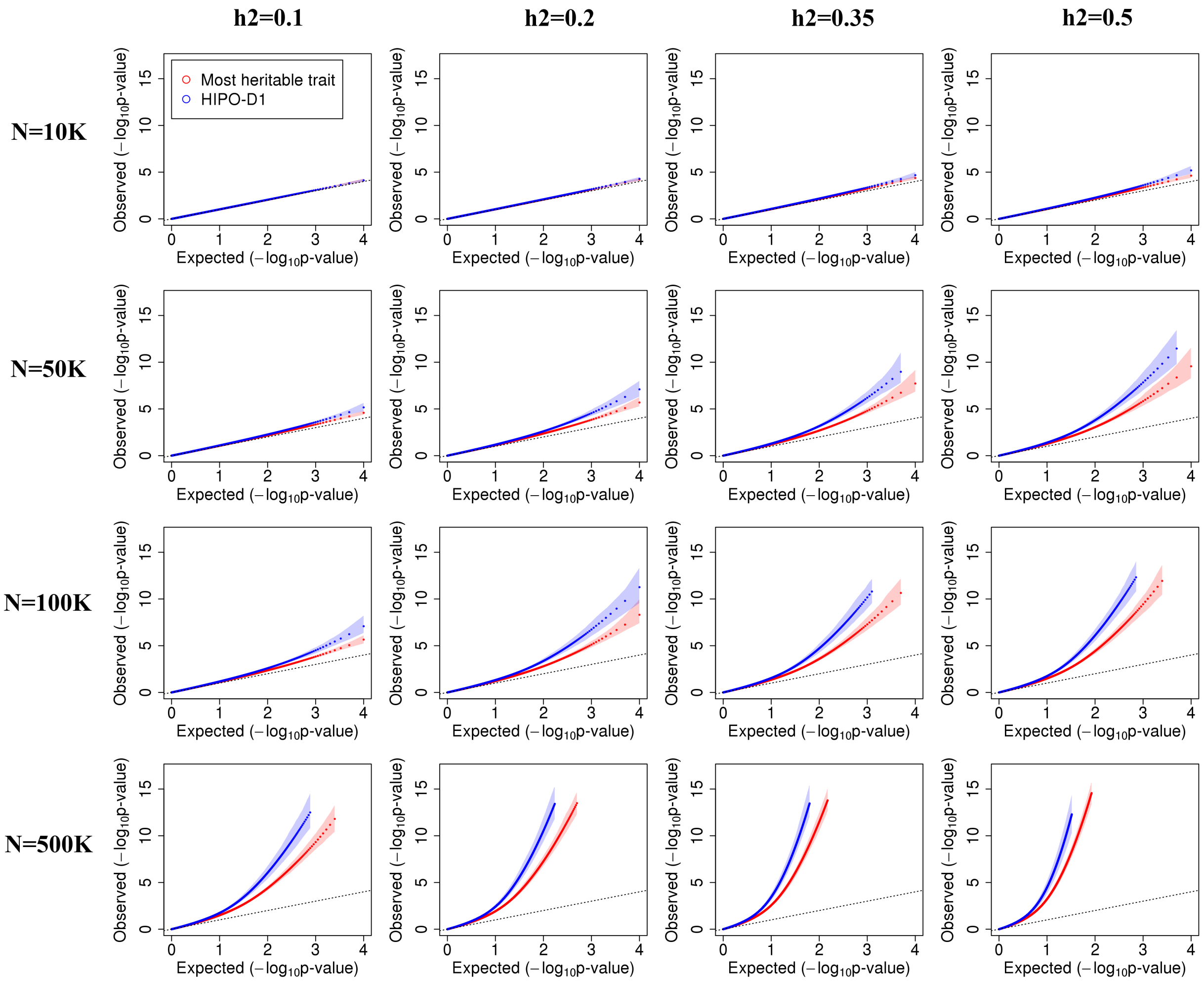

Supplement: S3 Fig — (PDF) [file pgen.1007549.s022.pdf]
