# Supplementary material for: Heritability informed power optimization (HIPO) leads to enhanced detection of genetic associations across multiple traits
Source: PLoS Genet. 2018 Oct 5;14(10):e1007549. doi: 10.1371/journal.pgen.1007549 (PMC6192650; doi:10.1371/journal.pgen.1007549)

S6 Fig. Venn diagram for the number of associated loci identified by individual trait analysis of psychiatric diseases, HIPO-D1 and meta-analysis. Independent SNPs are identified through LD-pruning with r^2 threshold of 0.1 and pruned SNPs were assumed to represent independent loci if they are >0.5Mb apart.

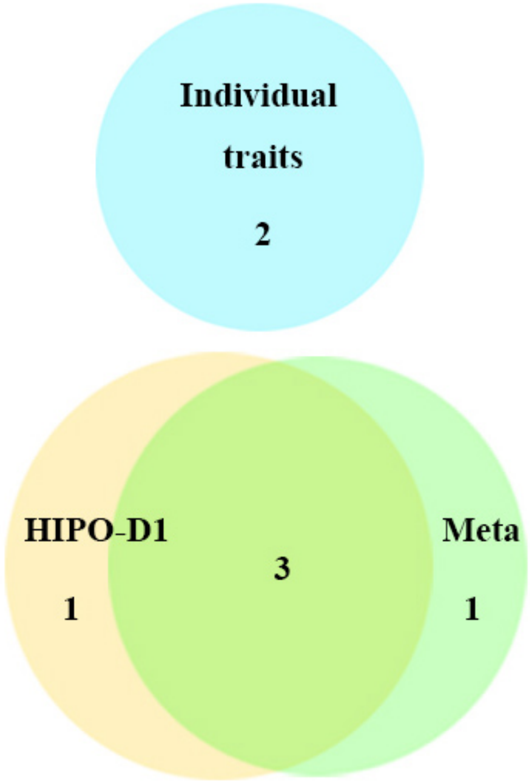

Supplement: S6 Fig — Independent SNPs are identified through LD-pruning with r2 threshold of 0.1 and pruned SNPs were assumed to represent independent loci if they are >0.5Mb apart. (PDF) [file pgen.1007549.s025.pdf]
